# The Importance of Being in Touch

**DOI:** 10.3389/fneur.2021.646640

**Published:** 2021-05-14

**Authors:** James R. Lackner

**Affiliations:** Ashton Graybiel Spatial Orientation Laboratory, Brandeis University, Waltham, MA, United States

**Keywords:** non-orientation, dynamic balance, position cues, path integration, vestibular loss, velocity storage, spatial orientation, vehicle control

## Abstract

This paper describes a series of studies resulting from the finding that when free floating in weightless conditions with eyes closed, all sense of one's spatial orientation with respect to the aircraft can be lost. But, a touch of the hand to the enclosure restores the sense of spatial anchoring within the environment. This observation led to the exploration of how light touch of the hand can stabilize postural control on Earth even in individuals lacking vestibular function, and can override the effect of otherwise destabilizing tonic vibration reflexes in leg muscles. Such haptic stabilization appears to represent a long loop cortical reflex with contact cues at the hand phase leading EMG activity in leg muscles, which change the center of pressure at the feet to counteract body sway. Experiments on dynamic control of balance in a device programmed to exhibit inverted pendulum behavior about different axes and planes of rotation revealed that the direction of gravity not the direction of balance influences the perceived upright. Active control does not improve the accuracy of indicating the upright vs. passive exposure. In the absence of position dependent gravity shear forces on the otolith organs and body surface, drifting and loss of control soon result and subjects are unaware of their ongoing spatial position. There is a failure of dynamic path integration of the semicircular canal signals, such as occurs in weightless conditions.

## Introduction

The studies described below had an unexpected starting point. I was working with Ashton Graybiel to determine the etiological factors causing motion sickness in the weightless conditions of orbital space flight. We were looking at the provocativeness of different types of head movements in the weightless and high g force phases of parabolic flight maneuvers. During the weightless phases, I would often free float to observe that subjects were carrying out their head movements appropriately. One day when free floating without any contact with the aircraft, when I closed my eyes, within seconds my sense of orientation to the aircraft disappeared. I could sense the relative configuration of my body, and cognitively I knew my spatial position in relation to the fuselage of the aircraft; but, I no longer felt being in any orientation. I had lost the sense of spatial anchoring that we normally have on Earth to our spatial context. However, when I touched the wall of the aircraft with one hand, the sense of my spatial position within the aircraft was restored. On removing hand contact, within a second or two, I was again anchorless. I was not spatially disoriented, instead I was “non-oriented.” We confirmed these observations with multiple subjects in later parabolic flight studies (1, 2).

**Figure 1:**
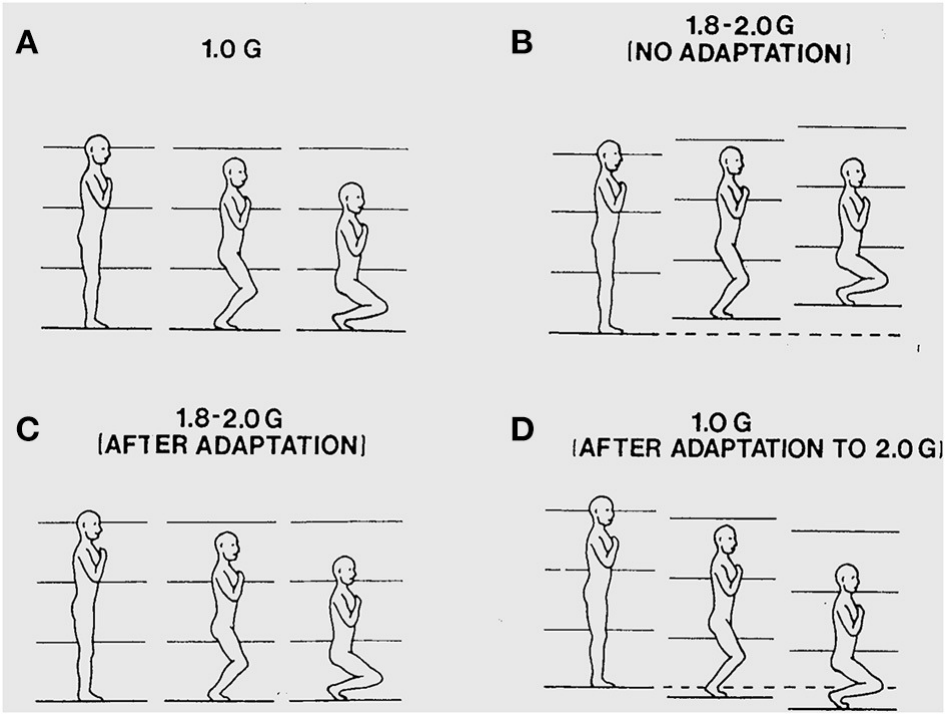
**(A)** Illustration of a deep knee bend made in 1 g. The surface of support and the visual surroundings are felt and seen to remain stationary as the body is lowered. **(B)** During a deep knee bend made during initial exposure to 1.8 g, it feels as if the knees have flexed too rapidly and the aircraft is seen and felt to displace upwards under the feet causing too rapid flexion of the knees. **(C)** Following about 50 deep knee bends made over subsequent parabolas, the deep knee bends again feel normal and the aircraft is seen and felt to be stable again as the body is lowered. **(D)** Following adaptation to 1.8 g, the initial deep knee bends made during 1 g straight-and-level flight again seem abnormal with the body seeming to move downward too slowly because the aircraft seems to move downward slowing the flexing of the legs.

I also found that when I walked on the deck of the aircraft during the high g phases of parabolic flight, it seemed unstable and my movements felt abnormal. I probed this instability by doing shallow deep knee bends during exposure to 1.8 g acceleration levels. I found that this elicited powerful visual and postural illusions. During the body lowering phase, it would seem as if my body had moved downward too rapidly in relation to the deck of the aircraft and that simultaneously the aircraft had moved vertically upward causing unexpectedly rapid bending of my knees. The apparent upward motion of the aircraft seemed greater when I closed my eyes. After I made repeated deep knee bends with eyes open during subsequent parabolas, adaptation occurred and the illusions abated. But, then, on return to 1 g conditions in straight and level flight, deep knee bends elicited illusions of opposite sign that then gradually abated with additional deep knee bends. Graybiel and I systematically replicated these observations experimentally. The results are illustrated in Figure 1 and show the initial illusory movements before adaptation and the aftereffects experienced upon returning to 1 g conditions after having adapted to 1.8 g. These findings indicate that sensory-motor control and perception of our body movements are dynamically adjusted to the force background of Earth gravity (3).

In later parabolic flight studies, Paul DiZio and I found out that if we made finger contact with a nearby surface when making deep knee bends during exposure to 1.8 g, the illusory visual and postural motions normally elicited were suppressed. The illusions returned as soon as finger contact was broken (2). These two sets of findings were critical: 1) loss of a sense of orientation when free floating with eyes closed that is restored by hand contact, and 2) illusions of self-motion and aircraft motion elicited during deep knee bends in a 1.8 g force background that are eliminated by hand contact with an aircraft-fixed surface. They led us to explore whether light touch contact might stabilize balance during upright stance under normal 1 g conditions.

## Light Touch Stabilization of Postural Control

In our first study, we measured body sway parameters for one-legged stance with and without visual cues, and with light touch or force touch of the index finger of the right hand with a laterally placed force plate. We limited light touch to a maximum of 1 N (<100 g) because biomechanical modeling of the subject's posture and arm configuration and applied finger tip forces showed this would be mechanically inadequate to attenuate body sway. No-touch conditions were included in addition to the light-touch and force-touch (ad lib as much force as the subject desired). The results showed that touch of the finger had a major effect (4). Subjects in their light-touch conditions were much more stable than in the no-touch conditions. Moreover, they spontaneously adopted an average contact force of ≈40 g. This value is interesting because Johansson and Westling earlier had shown that variations about this level lead to the largest modulation of firing activity in the tactile receptors in the fingertips of the thumb and index finger that are involved in the control of precision grip. During precision grip when a held object begins to slip, a long loop cortical reflex is elicited that adjusts grip forces within 125 ms (less than a voluntary reaction time) to stabilize the object (5, 6).

**Figure 2:**
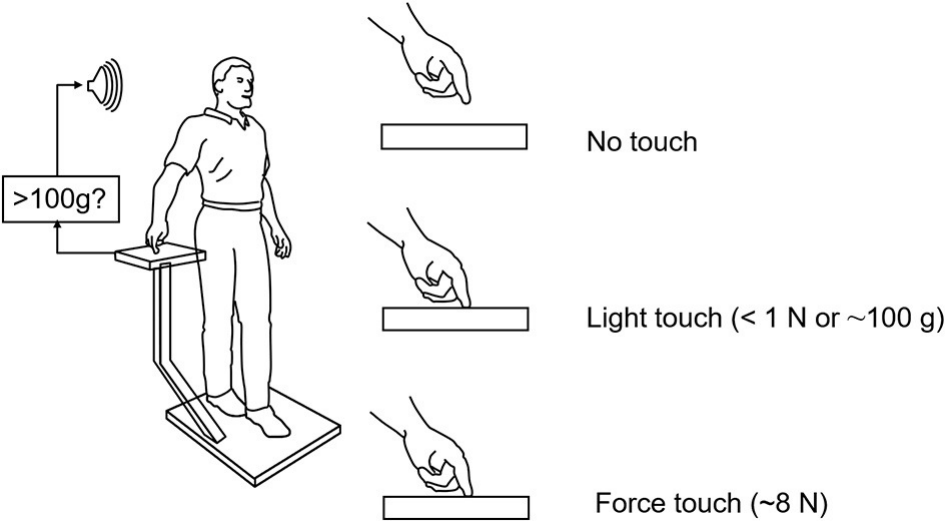
Typical test situation for our light-touch stabilization of posture studies. The alarm sounds when the force on the finger touch plate exceeds 1 N (≈103 g). Subjects in practice never reach this force level in their experimental trials. The alarm is off during force touch trials.

Force levels subjects adopted for light touch and ad lib force touch were ≈0.4 N (40 g) and 5–8 N, respectively. Light touch of the hand with vision stabilized the body somewhat more than with vision alone. The applied forces in the ad lib fingertip force condition were adequate to allow some mechanical stabilization according to our mathematical modeling but not to the extent actually observed. These observations together led us to hypothesize that light touch stabilization was the result of a long loop cortical reflex with the finger and the feet providing the contact surfaces of a pincer grip, with the feet serving as the “thumb” of the pincer (4). The results of this early study led us to explore the range of conditions under which non-supportive light touch of the hand can influence postural control. Only a sub-set of these studies can be described here. Figure 2 illustrates the typical test configuration. Test subjects would stand on a force plate in a heel to toe sharpened Romberg stance. A stand with a force plate was positioned to the subject's right side so that contact could be made with the right index finger in trials involving finger contact. In light-touch trials, an alarm would be sounded were the subject to apply more than 100 g to the touch surface. Before experimental data were collected, subjects would press on the plate to see how much force was necessary to trigger the alarm. Measures included mean sway amplitude, mean sway velocity, and power spectral density plots of sway as well as mean finger applied forces. All conditions referred to below as being different are statistically different by at least *p* < 0.01. The studies described below include: EMG evidence for light touch stabilization representing a long loop cortical reflex, finger force levels adequate for stabilization, time course of stabilization following finger contact, resistance to perturbations, entrainment of sway to a moving contact surface, and stabilization of balance of individuals without functioning labyrinths.

### Light Touch Stabilization of Posture Represents a Long Loop Cortical Reflex

**Figure 3:**
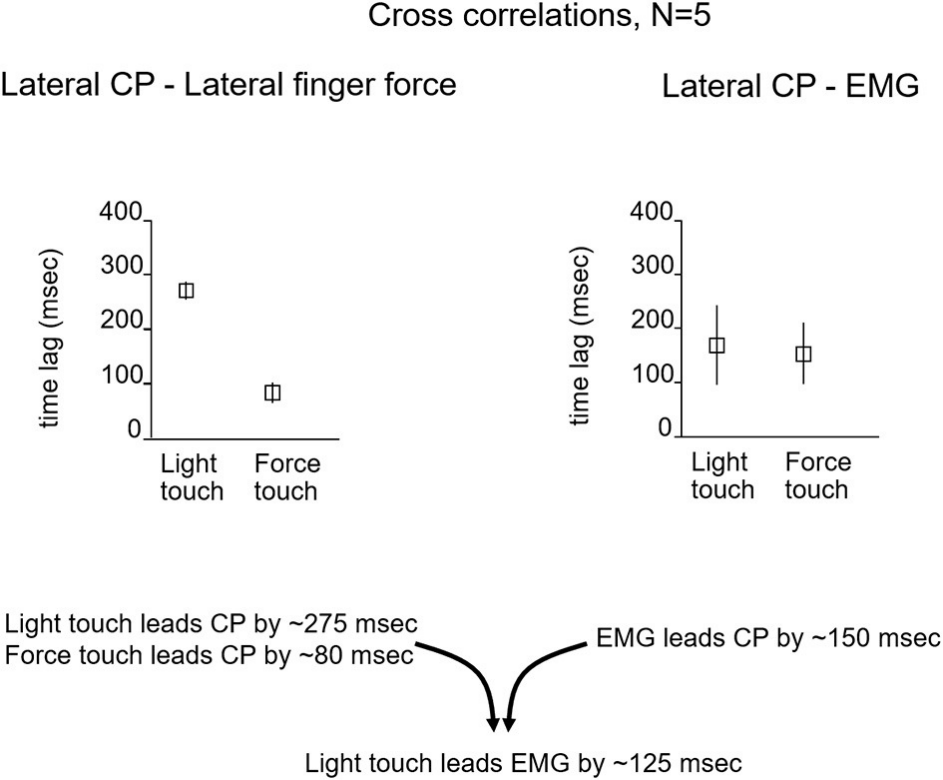
The relationship between the lateral CP and fingertip force changes and EMG activity that leads to CP changes. For light-touch, force changes at the fingertip lead EMG activity by 125 ms and CP changes by ≈275 ms. The EMG reflects muscle activation that 150 ms later alters the CP to counteract body sway. With force touch, changes in applied finger force lead CP changes by ≈80 ms, indicating some mechanical stabilization by the finger force level.

To evaluate the possibility of a long-loop cortical reflex being involved, John Jeka and I looked at the relationship between force contact changes during light touch of the fingertip, the center of pressure (CP) under the two feet, and EMG activity in the peroneus longus muscles. The subjects (*N* = 5) were blindfolded and in a heel-to-toe (sharpened Romberg) stance. Force changes at the fingertip during light touch led by 125 ms the EMG activity in the peroneus muscles that 150 ms later produced changes in the CP to attenuate sway as shown in Figure 3. This pattern persisted throughout the balance task and was analogous to the long loop cortical reflexes modulating precision grip control that are elicited when an object held in a pincer grip begins to slip. The difference in force generation latency between precision grip adjustments and postural control is related to the longer latency to control the mass of the body vs. that of the fingers (7, 8). Force touch led CP changes by 80 ms indicating that the force touch was mechanically adequate to attenuate sway.

### Finger Contact Thresholds for Touch Stabilization of Postural Sway

**Figure 4:**
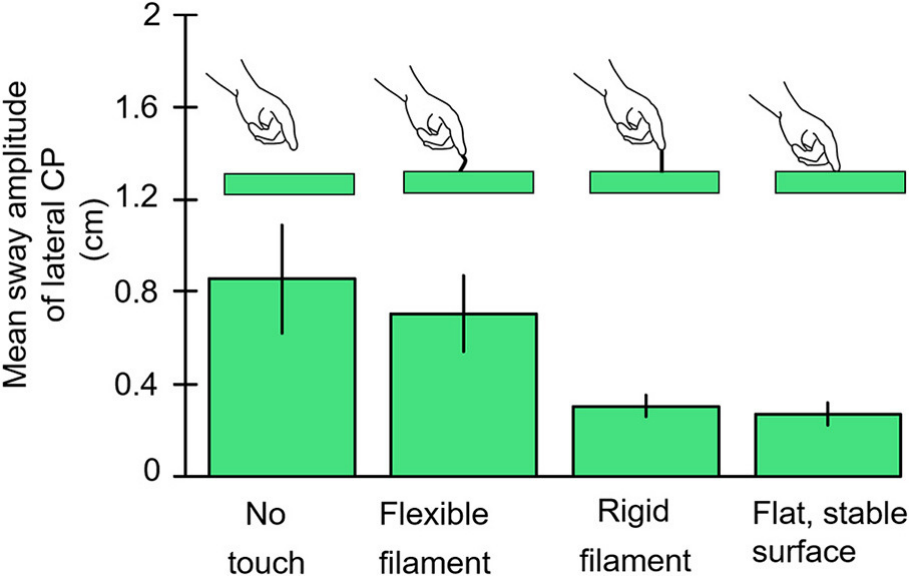
The results are illustrated for the no-contact, von Frey Filament with 10 g bending resistance, rigid metal filament, and light-touch finger conditions. The von Frey filaments with larger bending constants are not shown but all attenuate sway more than the 10 g filament. The rigid filament and finger touch attenuate CP mean sway amplitude more than other conditions.

Our goal was to determine the lowest level of force contact with the right index finger that would provide postural stabilization. Subjects stood with eyes closed, heel-to-toe, on a force plate while holding their right hand over a laterally placed touch plate. Von Frey filaments that are used to test tactile sensitivity in the clinic (9) or a rigid metal filament 1 mm in diameter would be mounted vertically on the hand touch plate. The Von Frey filaments had buckling values of 10, 35, and 85 g. Conditions were run in which the subjects touched the Von Frey filaments with and without enough force to bend the filaments, made finger contact with a flat surface, or held their finger as steady as possible just above the touch plate. Figure 4 illustrates the test results for no-touch condition, touching the 10 g filament, the rigid metal filament, and the flat surface, the latter two conditions were most effective in attenuating body sway and applied force levels hovered about 40 g. Holding the finger as steady as possible in imagined contact with a location just above the touch plate was the least stable condition. The Von Frey filaments all attenuated sway relative to the no-touch imagined contact condition. The non-bent filaments reduced sway more than the bent filaments of the same diameter. The bent filaments reduced sway magnitude such that sway never exceeded the range that would have led to a shift of the filament's contact point with the fingertip. Force levels of 10 g on our most slender Von Frey filament produced significant sway attenuation relative to the imagined contact condition. In all touch conditions, force changes at the finger led changes in CP by ≈300 ms (10).

### Time Course of Postural Stabilization After Fingertip Contact

**Figure 5:**
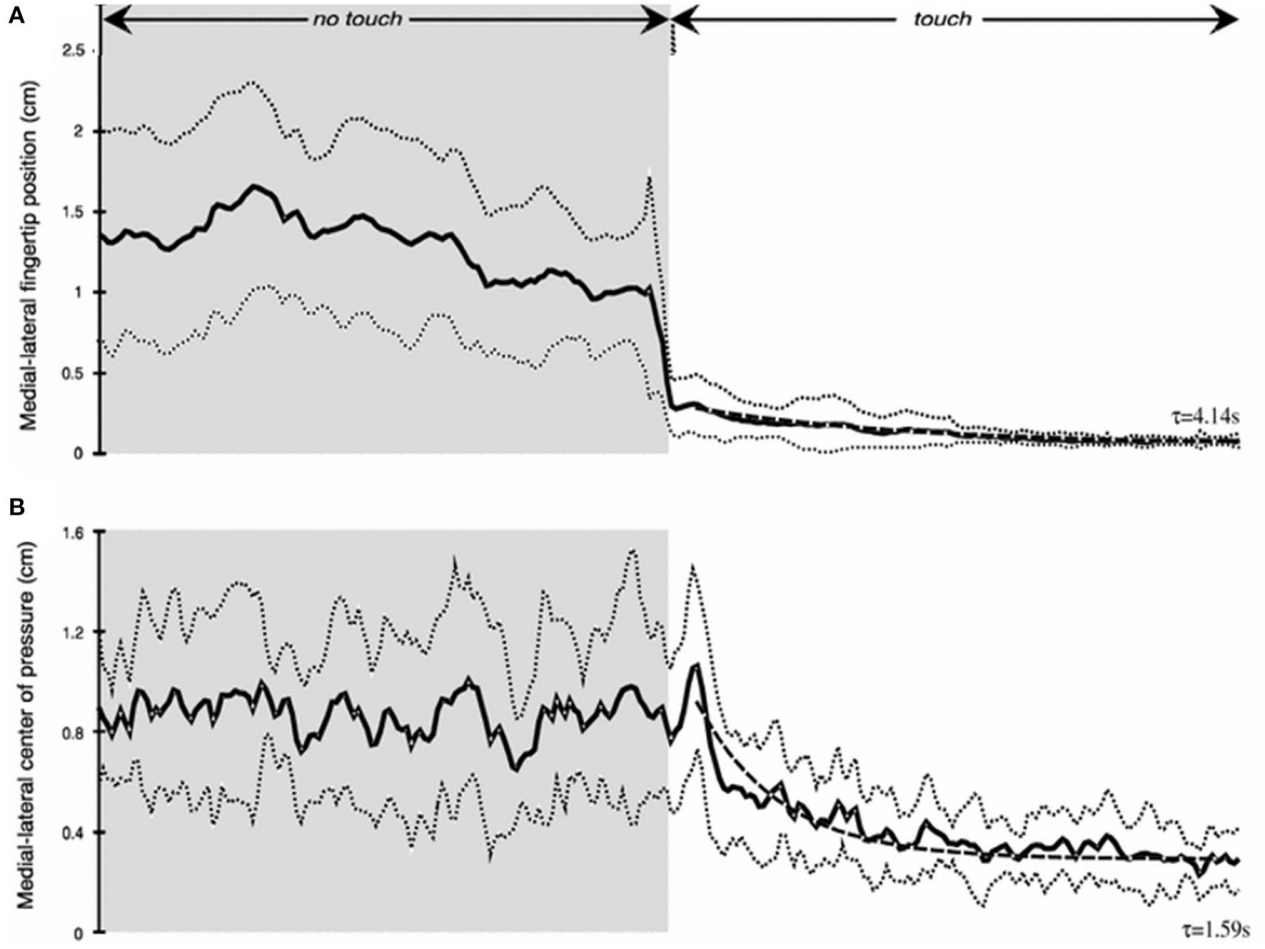
Time course of haptic stimulation after finger contact is made ≈ 12 s into 25 s long trial. **(A)** shows medial-lateral finger position (cm) and **(B)** medial-lateral center of pressure (cm). Note rapid decrease in sway even prior to full finger stabilization.

As our studies of haptic stabilization progressed we began thinking about potential haptic aids for enhancing the balance control of individuals at risk for falling. A key issue was how long would it take after touch contact was made for stabilization to begin. We tested blindfolded subjects in the heel-to-toe stance. They started a trial with their right index finger just above the laterally positioned touch force plate. Half-way through 25 s long trials they were cued to lower their finger to make contact. We found that the fingertip became fully settled on the plate to apply a force of ≈0.4 N with a time constant of ≈4 s. Mean sway amplitude of the body upon finger contact decreased by 50% with a time constant of 1.6 s (Figure 5). Importantly, within 500 ms after initial finger contact, correlated changes in the CP began to lag fingertip force fluctuations by 275–300 ms, even though the finger was not yet stabilized in position (11).

### Tactile Suppression of Postural Destabilization Induced by Tonic Vibration Reflexes

We also wondered whether an haptic cue from the hand would enhance postural stability in the face of perturbations. Our approach was to make use of tonic vibration reflexes (TVRs). When a skeletal muscle's tendon is stimulated using a physiotherapy vibrator (circa 100–120 Hz), muscle spindle receptors are activated and elicit a reflexive contraction of the vibrated muscle (12, 13). Eklund (14) had induced backward sway and loss of balance in standing subjects by vibrating their Achilles tendons. We wondered whether light finger contact with a stationary surface could overcome the destabilizing effects of leg muscle vibration and also whether vibrating the biceps muscle of the right arm of subjects making light touch with their right index finger would destabilize them.

**Figure 6:**
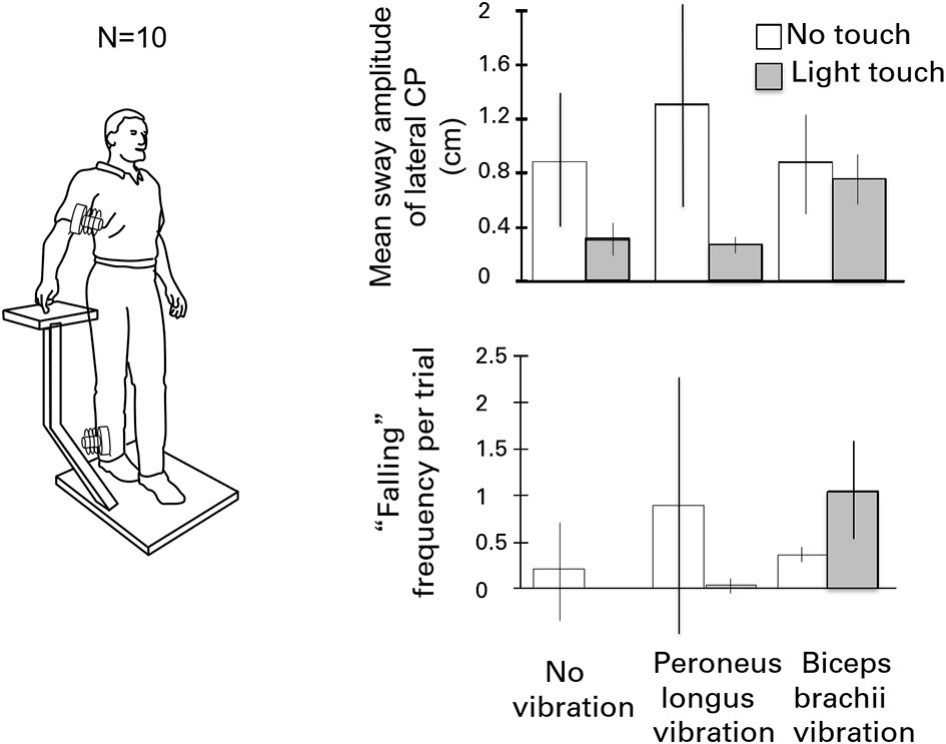
The effects of eliciting tonic vibration reflexes in the peroneus longus muscle of the standing subject's right leg or in the biceps of the right arm are shown for the different touch conditions. Vibrating the peroneus longus destabilizes subjects and can evoke falling. Light touch fully eliminates the effect. By contrast, during light-touch, biceps vibration is destabilizing and can induce falling or protective stepping.

Figure 6 illustrates the test conditions and summarizes the experimental findings. The leg vibration conditions included: no-touch and no-vibration, touch and no-vibration, no-touch and vibration, touch and vibration. Subjects had their eyes closed during the trials. Vibration involved stimulation of the right peroneus brevis and longus muscles at 120 Hz to elicit tonic vibration reflexes. The results were striking. The touch and vibration and touch and no-vibration conditions were not significantly different for any sway measure and were vastly superior to the no-touch and no-vibration and no-touch and vibration conditions. Importantly, in the touch and vibration condition, many of the subjects reported that it felt as though the vibrator on their leg had not been turned on. By contrast, in the no-touch and vibration condition, subjects often had to take a protective step to avoid falling (15).

In the parallel set of conditions for the influence of vibration of the biceps of the right arm on postural stability, the no-touch with vibration and touch with vibration conditions did not differ in mean sway amplitude from the control condition of no touch and no vibration. In addition, the incidence of “falling” or making a protective step, was increased when the biceps of the arm of the touching finger was vibrated to cause reflexive contraction. The forearm motion elicited by biceps vibration led to postural shifts to null the finger's displacement, sometimes eliciting lateral falling and stepping.

### Entrainment of Sway to a Moving Touch Surface

**Figure 7:**
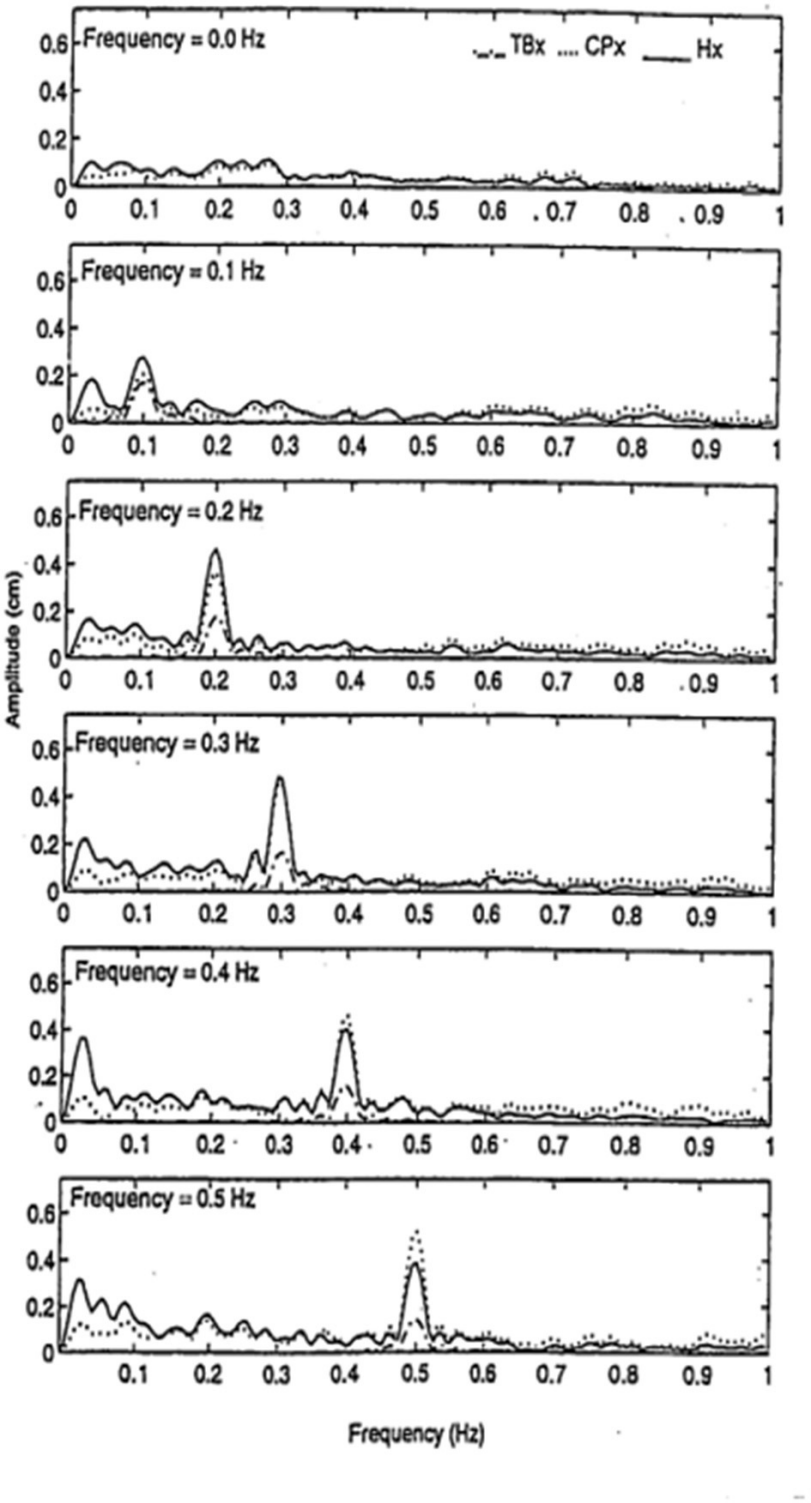
Entrainment of body sway to cyclical lateral oscillations of the touch bar at frequencies of 0, 0.1, 0.2, 0.3, 0.4, and 0.5 Hz. TB_x_ = lateral motion (≈4 mm amplitude) of touch bar, CP_x_ = lateral motion of the center of pressure, and H_x_ = lateral head motion. Subjects (*n* = 5) show entrainment of head and center of pressure to the touch bar frequency of oscillation.

We had found that finger contact with a stationary surface was a powerful stabilizer of posture, but we wondered how contact with a moving surface would affect sway when the subjects did not know the surface could move. We had subjects, with eyes closed, stand in a sharpened Romberg stance and make finger contact with a laterally placed surface at <100 g force. In a control condition the surface was stationary, in five other conditions it oscillated laterally at 0.1, 0.2, 0.3, 0.4, or 0.5 Hz at an amplitude of ≈4 mm. As shown in Figure 7, spectral analyses of head and body sway in relation to touch surface displacement frequency showed a close coupling with amplitude peaks at the frequency of motion of the touch surface. Touch forces applied to the surface were always <100 g and were comparable across all six test conditions. Modeling the postural control system as a second-order linear dynamical system led us to conclude that the velocity of the signal at the fingertip was key to the sway coupling (16). Jeka et al. (17) have further explored this coupling in conditions where stimulus velocity was maintained constant across different frequencies and found coupling to both displacement and velocity.

### Haptic Stabilization of Posture in Individuals Without Vestibular Function

We were especially interested in whether people with loss of vestibular function could be aided by haptic cues. We knew that such individuals typically can stand heel-to-toe with eyes closed for only a few seconds without having to take a protective step. To evaluate the role of haptic cues, we tested five labyrinthine loss subjects. They ranged in age from 52 to 70 years, mean 59 years. Several had bilateral vestibular loss from streptomycin poisoning, another from an autoimmune disease, and one from progressive neural degeneration of unidentified etiology. All had been evaluated at the Vestibular Testing Laboratory at the Massachusetts Eye and Ear Infirmary in Boston. They were selected from a large group of patients with vestibular loss because they fell at the lowest end of the vestibular loss category of performance on semicircular canal, otolith, vestibulo-ocular, dynamic posturography, and visual-vestibular interaction tests. Their gains on the sinusoidal vertical axis rotation test (0.05 Hz) ranged from 0.017 to 0.169, with an average of 0.057. Responses to caloric irrigation were absent, as were responses to the head thrust test. Five age and sex matched control subjects were recruited from members of the Brandeis University staff. Their performance on all tests of vestibular function and balance were within the normal range.

**Figure 8:**
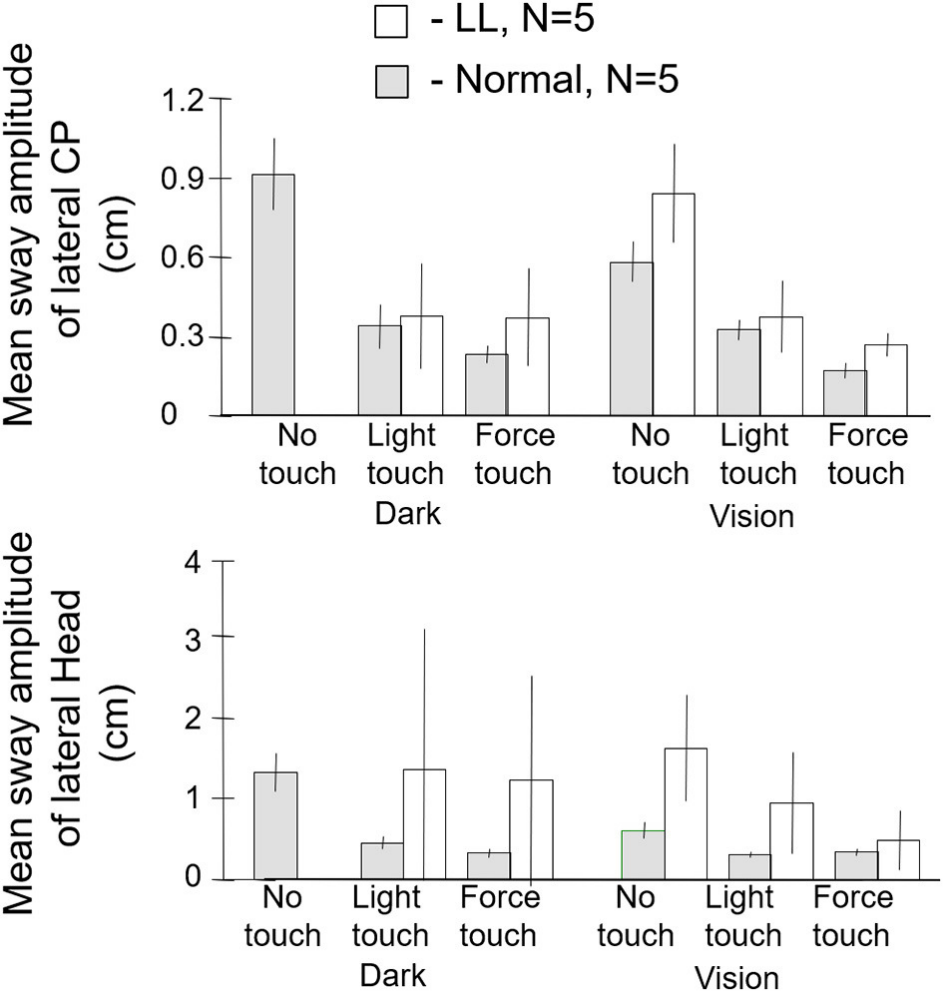
Mean sway amplitude of center of pressure (CP), top panel, and of head, bottom panel of labyrinthine loss (LL), *N* = 5 and normal subjects, *N* = 5. There are no entries in the dark no-touch conditions for the LL subjects because they lose balance within several seconds. With light touch in the dark, the LL subjects have a significantly lower CP mean sway amplitude than the normal subjects in their no-touch vision condition.

The subjects were tested in the sharpened Romberg stance under eyes open and eyes closed conditions for each of three fingertip conditions: no contact, light contact (<100 g) with a laterally place surface, and ad lib force level contact. Safety railings surrounded the test setup. The control subjects did not lose balance in any test condition. All of the labyrinthine loss subjects (*N* = 5) lost balance within 5 s in the eyes closed no-touch condition and had to grasp the safety railings or take a protective step. By contrast, as shown in Figure 8, when allowed light touch contact, they were more stable with eyes closed than the normal subjects were with eyes closed and no finger contact. With eyes open and no touch, the labyrinthine loss subjects typically had to touch the safety railings several times for support during the 25 s duration trials. However, with eyes closed and light touch, they were more stable than they were in the eyes open no-touch condition. Both groups of subjects were most stable with eyes open and light touch. The ad lib touch force condition usually conferred the greatest stability for both groups. In the light-touch conditions, for all subjects force changes at the fingertip led changes in the CP by 250–300 ms (18). There is no entry in Figure 8 for the labyrinthine loss subjects for the eyes closed, no-touch condition because they could not perform the task.

## Alternative Interpretations

Our light touch studies elicited considerable interest as well as alternative interpretations. These included proposals that posture was stabilized to allow precise finger contact rather than the reverse (19). Other investigators provided evidence that passive touch was as effective as active touch in stabilizing posture (20, 21). We recently have completed work showing that when passive touch (100 g) is applied to the shoulder by a lever device attached to a force plate so that the instant of contact and change in contact force can be determined, within 500 ms, the force changes at the shoulder elicited by body sway lead by ≈300 ms changes in the CP to counteract the sway. Put differently, the cortical long loop reflex to stabilize sway is not confined to the finger but can be evoked by other parts of the body as well. When the passive probe is lifted from the shoulder, within 1 s postural sway increases despite attempts to be as stable as possible (Lackner et al., in preparation). Another earlier study manipulating stance configuration and the location of the touched surface showed that contact in the direction of body sway maximizes stabilization (22). Immobilizing the entire arm of the touching finger using a “Freedom Gunslinger” splint (AliMed, Dedham, MA) did not prevent contact of its finger with a stable surface from attenuating sway (23). Bove et al. (24) have shown that light touch suppresses the disorientation evoked by neck muscle vibration, just as it suppresses the effects of leg muscle vibration.

The touch stabilization paradigm has been extended by Wing and Johanssen in a number of very imaginative ways of potential use for rehabilitation and fall prevention. They have shown that interpersonal touch at very low force levels can be helpful in stabilizing older people's posture and gait (25–29). Sawers and Ting have shown how small inter-person forces can be used to communicate goals and actions between individuals but also how they can be incorporated in the design of rehabilitation robots (30, 31).

## Central Contributions to Balance Control

The postural stabilization experienced by the labyrinthine loss subjects when provided light touch of the hand with a stable surface was an important finding for us, but it also presented a conundrum. In posture studies, the CP under the feet during quiet stance is often used as a proxy for the location of the body center of mass (CM), and the soles of the feet can be thought of as providing a map of the projection of the CM (32, 33). Normal subjects during quiet stance with eyes closed sway at velocities and magnitudes that can be near or just above vestibular detection thresholds. Why then are labyrinthine loss subjects unable to stand heel to toe for more than a few seconds when they close their eyes? Presumably, they have the same somatosensory cues from their feet about CM position as normal subjects do. We knew from our light-finger touch studies that they benefitted as much from sensory cues from the hand as normal subjects. Why then did not the soles of their feet convey similar benefit?

Karmali et al. have recently conducted the most comprehensive assessments of semicircular canal and otolith detection thresholds for all axes of rotation and translation yet carried out (34). Their apparatus was a 6 DOF motorized platform that subjects were seated on in the dark. They measured the smallest motion that subjects could reliably detect. They then correlated these thresholds with a variety of postural tests carried out on the same subjects. A key finding was that lateral translation thresholds were correlated with medial-lateral CP sway during passive stance; but, the other thresholds were not. They conclude that vestibular noise contributes to the magnitude of lateral postural sway because their threshold measurements also represent the magnitude of sensory noise. From this perspective, labyrinthine loss subjects would be expected to have larger postural sway.

Stoffregen and Riccio have made the important point that during upright stance on a horizontal surface the direction of balance coincides with the direction of gravity (35, 36). They posed the possibility that the perceived vertical corresponds with the direction of balance not that of gravity. To explore this idea they used a device programmed to behave like an inverted pendulum in roll. A subject would sit in it and use a joystick to set it to the “upright.” This approach takes advantage of the fact that human passive stance is often modeled as a single link inverted pendulum (37), which is appropriate for their test situation. The novel feature of their device was that its direction of balance (DOB) could be offset from the direction of gravity (DOG). When the DOB is offset from that of the DOG, this means that when the device is at the DOG there is an acceleration driving it in the direction opposite the DOB. The DOB represents the position of dynamic equilibrium, rather than that of gravity as is normally the case in upright stance. In their studies, Stoffregen and Riccio had subjects set the device to the “upright” when its DOB corresponded with the DOG and when its DOB was displaced in the roll plane to the left or right of the DOG. With an offset DOB, in setting themselves to the upright their subjects maintained the average position of the device between the DOB and DOG. Consequently, it was concluded that the DOB influenced the perception of the upright and that it, rather than gravity, was key to the perceived upright (38).

**Figure 9:**
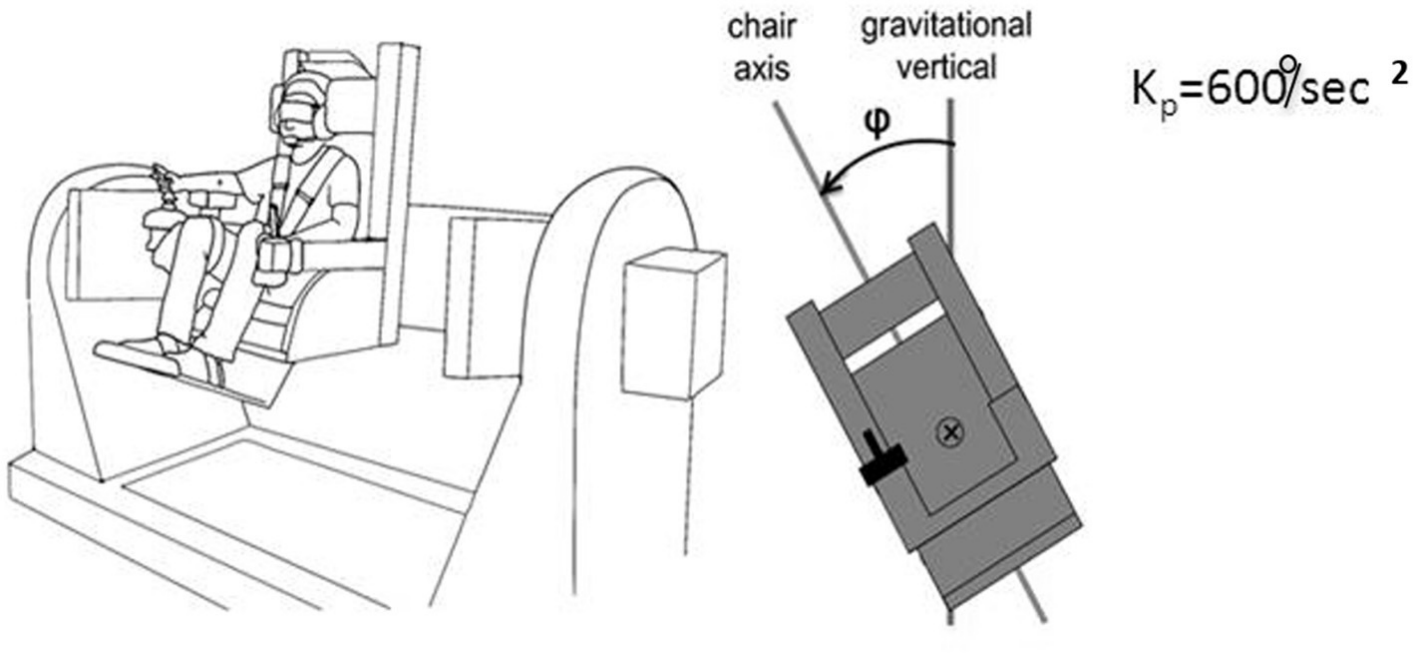
Illustration of the MARS configured for vertical plane roll balancing. The axis of roll motion is indicated by the ⊗ symbol, the deviation from the gravitational vertical is angle φ. Kp = 600°/s^2^ is the inverted pendulum constant.

We realized that a similar apparatus provided a means to separate peripheral mechanisms related to foot control in balance from central mechanisms, and could potentially provide insight into the conundrum we felt was posed by our labyrinthine loss subjects. “Peripheral” is meant here to include vestibulospinal reflexes and other leg muscle reflexes, as well as pressure distribution on the soles of the feet, the plantar map of the center of mass. This led us to develop a device that allowed us to look at more than one axis of rotation and plane of motion to assess central contributions to balance control. The advantages of our multiaxis rotation system (MARS) are that it can rotate about a pitch, roll, or yaw axis, singly or in combination, and that the plane of motion can be set to any desired angle in relation to the direction of gravity. Subjects seated in the MARS control it using a joystick. When subjects exceed ± 60° from the instructed goal heading they are regarded as having “crashed” and the MARS is reset to its original start position and the trial continues. An important feature of the MARS is that its motion profile as generated by a subject actively controlling it can be recorded and then played back to the same or a different subject who sits in it and uses joystick trigger presses to indicate when he or she is at the instructed goal direction. Figure 9 illustrates the MARS device configured for vertical roll plane balancing.

### Direction of Balance vs. Direction of Gravity in Perception of the Upright

**Figure 10:**
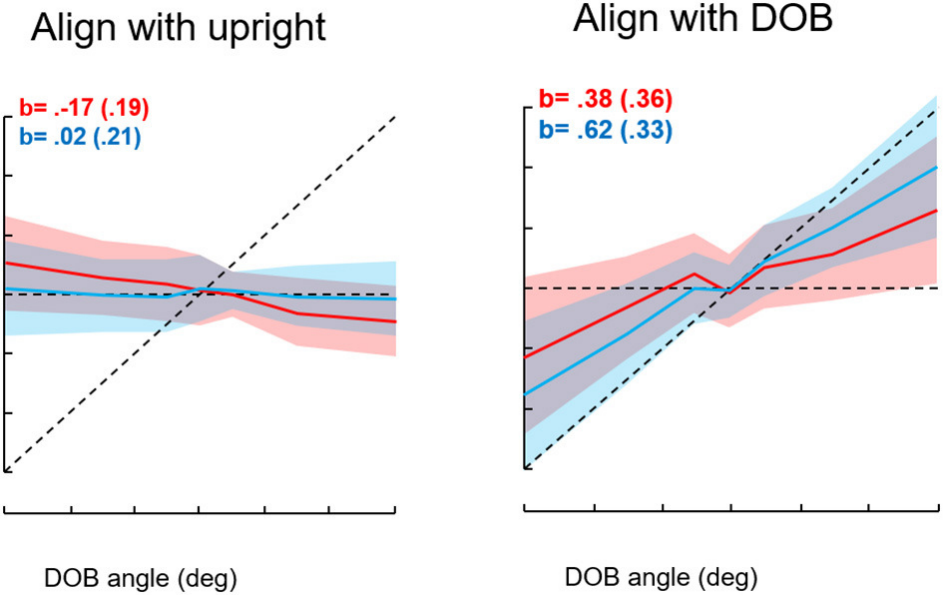
Results for the conditions “align with the upright” and “align with the direction of balance.” Seven directions of balance (DOB) were presented twice for each instruction type for 16 subjects. The DOBs were −30°, −15°, −5°, 0°, +5°, +15°, and +30°. Red entries represent achieved angles, blue represents indicated angles. The shading indicates standard deviations. The b entries represent slopes of MARS angles in degrees vs. DOB angles. The sign convention is minus entries represent rightward tilts of the subject and DOB, positive is leftward tilts. The intersection of the dotted horizontal and diagonal lines corresponds to 0°, the direction of gravity.

We first studied roll orientation to confirm the observations of Riccio et al. (38). In different trials, we asked blindfolded subjects to orient to the “upright,” the DOB, or the DOG. In addition, we instructed subjects to press the joystick trigger whenever they were at or just passing through the intended goal. We found that when the DOB was offset from the DOG, settings to the upright were on average displaced past the DOG away from the DOB. However, trigger presses to indicate the perceived upright or the DOG closely coincided with the DOG. Settings to the DOB were on average about midway between the DOB and DOG, but trigger presses to indicate the DOB were closer to it. Thus, as shown in Figure 10, there was a discrepancy between the average orientation of the subject in the MARS to the goal orientations and the subject's trigger presses. To understand why this could be the case we looked at the relationship among joystick control inputs, the ongoing position of the MARS, and the joystick trigger presses in relation to the goal orientation. This analysis resolved the paradox. With an offset DOB, subjects trying to set the apparatus to the perceived upright were being exposed to a unidirectional acceleration that increased in magnitude as they got close to the DOG. To orient to the DOG, subjects as they neared the DOG slowed the MARS motion and their trigger presses occurred when it was just at the DOG. They then eased off on the joystick and the MARS was again pushed away from the DOG in the direction opposite the DOB. In other words, the discrepancy relates to using a joystick to control the apparatus during exposure to an unidirectional acceleration that progressively increases near and beyond the DOG. To avoid going past the DOG in the direction of the DOB subjects try to reverse the device's direction just as they are nearing the DOG. Consequently, the average position of the device ends up being beyond the DOG and away from the DOB as a consequence of control rather than perceptual limitations (39). The direction of gravity, not that of balance, determines the perceived upright.

### Perception of the Upright Is Not Enhanced by Active Control

We were also interested in whether active control influenced the ability of subjects to distinguish between the DOG and DOB when the DOB was offset from the DOG. To test this we recorded the motion of the MARS when subjects were actively controlling it and then played it back to subjects who rode in it “passively” and pressed the joystick trigger when they felt that they were at their goal orientation. The goal was to indicate the DOB, the DOG, or the “upright” depending on the active subject's task during the profile that was being played back to them. The findings were unequivocal: the trigger presses of the passive subjects for each goal condition were not significantly different from those of the active subjects. Together these results mean that subjects balancing in the apparatus or being passively transported in it identify the perceived upright with the DOG and can accurately indicate it (39).

### Balancing With Diminished Gravity Dependent Positional Signals

**Figure 11:**
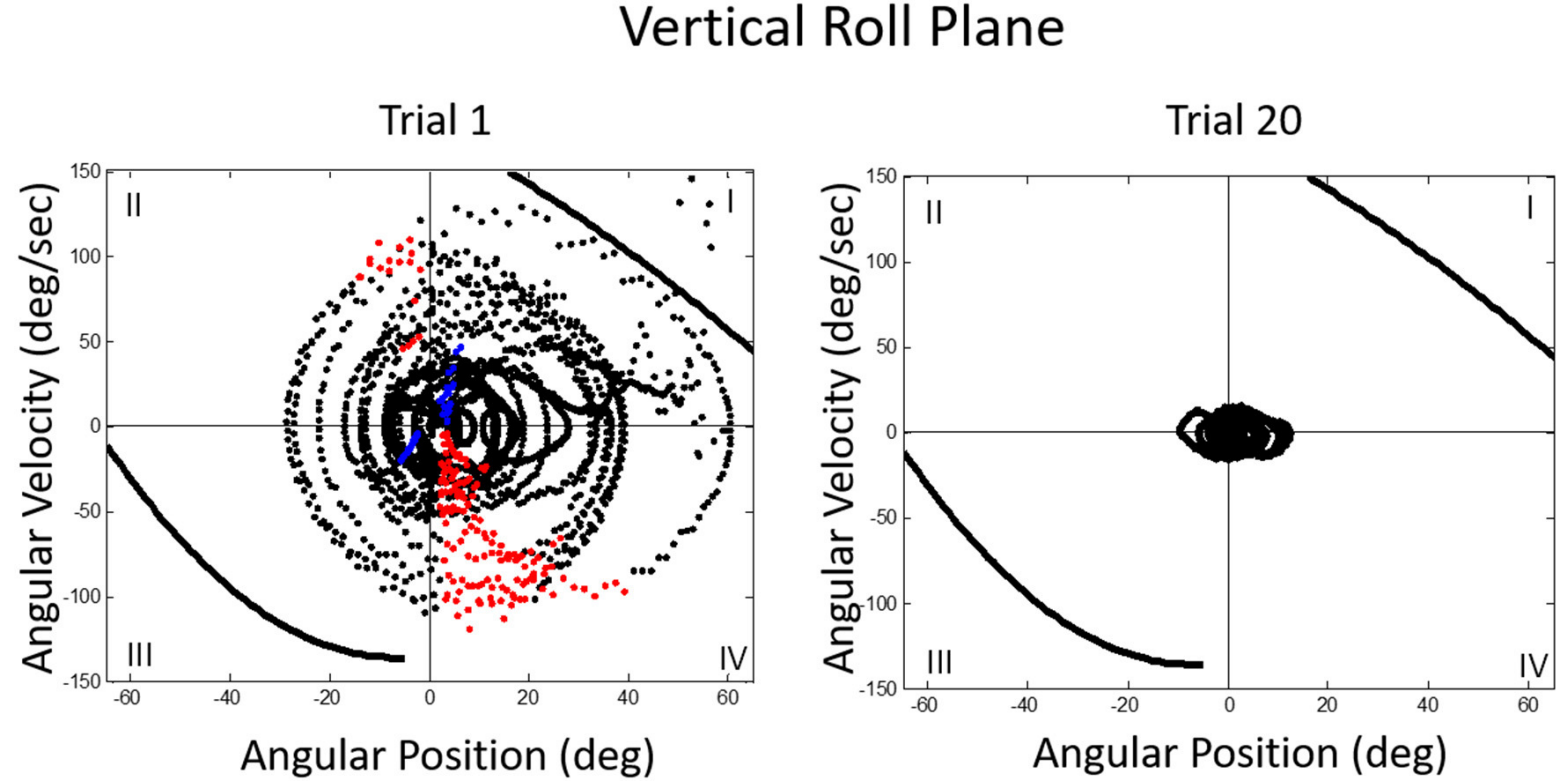
Velocity-position phase plots for a typical subject in vertical roll plane balancing. Solid lines in quadrants 1 and 3 represent crash boundaries where joystick commands cannot prevent the MARS from exceeding + or −60 degrees. Dots outside these lines represent crashes. The MARS is reset to the start position after a crash and the trial then continues. Performance is near perfect after 20 trials. The red dots represent anticipatory joystick commands that slow the MARS down as it approaches the balance point. Blue dots represent destabilizing joystick commands that accelerate the MARS in the direction of a crash boundary.

**Figure 12:**
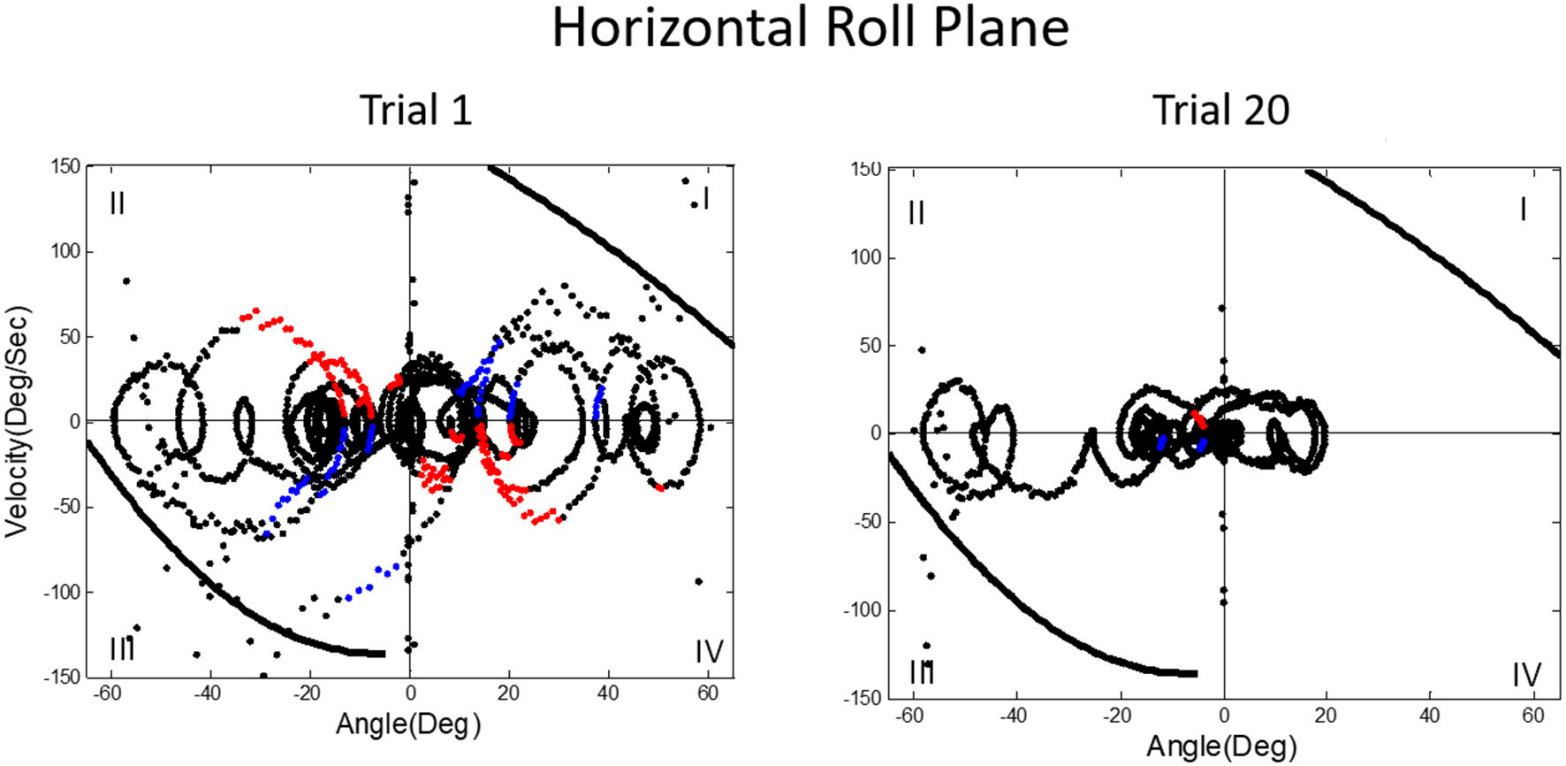
Velocity-position phase plots for a typical subject in horizontal roll plane balancing show cyclical patterns of looping and drifting. The drifting and looping are reduced but not eliminated over repeated trials. Crashes are decreased but not eliminated as shown by dots outside crash boundaries.

We then asked what happens to balance performance when the roll plane is tilted backwards from the vertical so that the contribution of the otolith organs is progressively diminished. We realized that this provided an analog of weightless conditions in which the otolith organs do not provide signals about changing body orientation, but the semicircular canals will still be activated by angular accelerations. In this experiment, subjects were orienting to and indicating the DOB, which if the roll plane were vertical would correspond with the DOG. We tested a range of tilt-back roll plane angles and performance remained unchanged until about 55°, after which it rapidly diminished, until at 90° tilt back subjects showed cyclical or looping patterns of drifting and frequent crashes. At this body orientation the utricular planes are near parallel with the DOG and the saccular planes are near perpendicular to it. Consequently, they no longer provide positional information about body location within the roll plane. This situation is an analog condition to that of labyrinthine-loss subjects who have to balance without otolith (and semi-circular canal) signals. Gravity dependent somatosensory cues about body position in roll are also minimized. In this circumstance, subjects exhibit drift, cyclical oscillations, and frequent crashes. They report not knowing which way they are moving and where they are in the roll plane. This is the case despite angular acceleration levels being well above measured semicircular canal thresholds (40). Figures 11, 12 illustrate vertical and horizontal roll plane performance, respectively.

### Asymmetric Transfer of Training Between Vertical and Horizontal Roll Plane Balancing

The decrement in roll balance control with the body supine was concerning given its implications for performance in space flight conditions. To see whether practice in balancing with gravity-dependent cues could enhance performance when balancing without them, we gave one group of subjects repeated trials of balancing the MARS in the vertical roll plane where they had otolith and somatosensory cues about body position. We then tested them to see whether they would show transfer of learning to balancing in a supine roll plane the next day. We exposed another group of subjects to supine roll plane balancing on Day 1, where they only had transient semicircular canal and tactile cues to rely on, and then on Day 2 had them balance in the upright roll plane. We then compared the two groups performances for vertical and supine roll plane balancing. The results were unexpected. Subjects who were tested in the vertical roll plane on Day 2 (after supine roll plane balancing on Day 1) showed significantly better performance on five key measures compared to subjects who had vertical roll plane balancing on Day 1, including control of MARS position and standard deviation, fewer destabilizing joystick commands leading to crashes, less cumulative drifting, and reduced joystick deflection magnitude. Thus, the subjects who had already experienced supine roll plane balancing showed substantive transfer to vertical roll plane balancing. By contrast, subjects who underwent supine roll plane balancing on Day 2 following vertical roll plane balancing on Day 1 performed no better on any measure than the subjects who balanced in supine roll on Day 1. Prior experience with vertical roll plane balancing had provided no benefit whatsoever (41).

On examining the detailed pattern of the experimental results, we realized that subjects initially exposed to upright roll could rely on both gravitationally dependent otolith and somatosensory signals to determine their orientation. Consequently, when exposed on Day 2 to supine roll where such cues were absent, they were in the same situation as subjects exposed to supine roll on Day 1. They had to rely on semicircular canal signals to orient and try to avoid crashes. Subjects exposed on Day 2 to upright roll had learned on Day 1 to rely on semicircular canal signals associated with joystick movements to try and avoid crashing during supine roll. From debriefings it became clear that subjects tested in supine roll on Day 1 had learned joystick strategies to avoid crashes. The strategies did not improve their sense of body position but the joystick strategies coupled with the addition of otolith and somatosensory cues about ongoing position benefited them when exposed to upright roll on Day 2.

### Training Strategies to Enhance Dynamic Balance Control in Supine Roll

We then asked whether we could train subjects to use strategies that would improve their ability to balance in the absence of position dependent gravity cues. Our approach was to expose subjects to vertical roll balancing with a DOB offset with respect to the DOG. Their goal was to orient to the DOB. They were repeatedly exposed to offset DOBs to the left or right of the DOG and, after each trial, were given verbal feedback about where the DOB had been and how well they had performed. We found that this approach helped them to learn strategies to home in on the DOB when they had ongoing otolith and somatosensory cues to their body orientation. After training in this fashion, when then exposed to supine roll balancing, all subjects showed performance improvements compared to their initial trials of exposure to supine roll prior to training. They had benefitted from the training but still had no firm sense of where the DOB was. They could not sense their actual position within the horizontal roll plane (42).

### Predicting Ultimate Performance Levels

A key focus of ongoing work is to see whether our training paradigms to enhance balance control under conditions of reduced positional cues can be translated to operational aerospace conditions by relating joystick control movements to the subject's current ongoing position, direction, and velocity in relation to the DOB. By looking at these parameters and using machine learning computational modeling, we have found that it is possible with 80% accuracy within an initial block of five 100 s duration trials to classify subjects as “proficient,” “average,” or “non-proficient” in terms of their ultimate performance level at the completion of 10 blocks of 5 trials (43). This classification capability is important for assessing performance in dynamic conditions involving vehicular control in aviation, e.g., helicopters, and situations involving precise maneuvering control, e.g., spacecraft docking conditions. It raises the possibility of identifying early on individuals training to be pilots who would benefit from individualized training protocols.

### Failure of Path Integration in Dynamic Balance Conditions Without Gravity Dependent Position Cues

**Figure 13:**
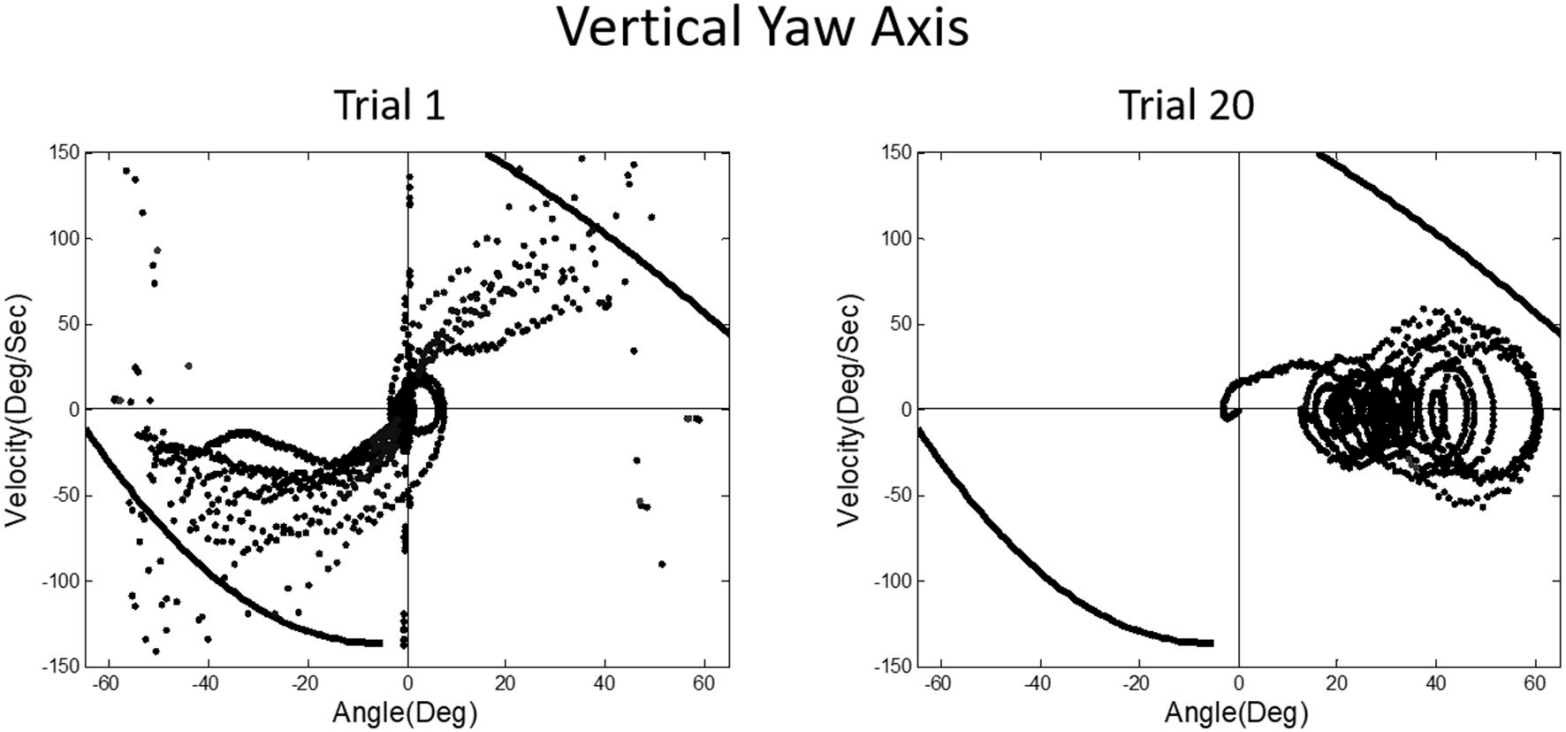
Velocity-position phase plots for vertical yaw axis balancing. The drifting and looping and crashes characteristic of horizontal roll plane balancing (Figure 12) are apparent for the yaw axis, where there are also no gravity dependent positional cues available about ongoing yaw rotational position. Subjects, as in horizontal roll, report being unable to sense their ongoing position relative to the direction of balance.

**Figure 14:**
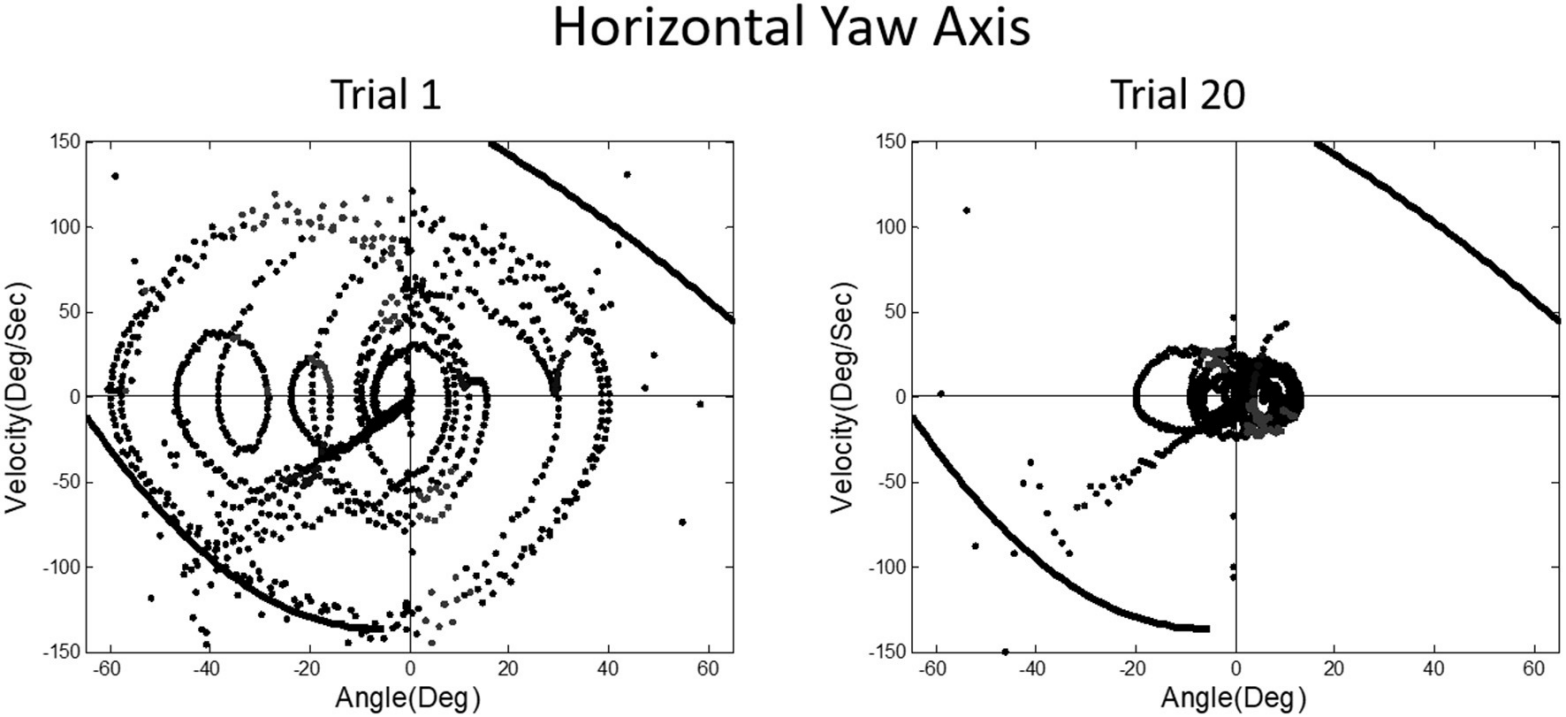
Position-velocity phase plots for a typical subject performing horizontal yaw axis dynamic balancing. In this situation, gravity dependent positional shear forces are present and learning is obvious from Trial 1 to 20, just as in vertical roll plane balancing shown in Figure 11.

A prominent feature of terrestrial life is path integration, keeping track of our ongoing position in relation to the environment. Path integration is often studied in the laboratory by exposing subjects seated upright in a rotating chair or robotic chair to angular displacements of various magnitudes and having them estimate their experienced displacements, using “look back” saccades or their feet to return the chair to its start positions. Typically such estimates are quite accurate and are thought to depend on integration of semicircular canal velocity signals to give a positional change (44–51). A key feature of our MARS device is that it can be programmed as an inverted pendulum about any axis. When we programmed it to exhibit inverted pendulum behavior about a vertical yaw axis, we found that subjects trying to orient to the DOB showed cyclical drifting and were unable to sense the DOB. The velocity-position phase plot for a typical subject is shown in Figure 13. By contrast, when the axis of yaw rotation was horizontal, subjects were able to sense and orient to the DOB, as shown in Figure 14.

In upright yaw, there are no position dependent gravity shear forces on the otolith organs and body surface; by contrast, in horizontal yaw balancing strong position dependent otolith and somatosensory cues are present. Thus, these results are exactly parallel to those for horizontal and vertical roll plane balancing, respectively (52). In the absence of position dependent shear forces on their body, subjects have to rely on semicircular canal signals and performance breaks down. The canal signals apparently cannot be accurately sequentially integrated to give a fiducial representation of ongoing body position. These results for yaw axis control were initially surprising given the work showing accurate path integration for single positional changes in upright yaw axis rotation.

### Resolving the Conundrum of Why Static Postural Control Is so Degraded in Vestibular Loss Subjects

The MARS experiments suggest that a normal subject's ability during quiet stance to sway near vestibular threshold levels for detecting linear or angular acceleration relates to the otolith organs providing a fiducial signal of head position with respect to gravity. Coupled with pressure information about the CP from the feet and proprioceptive information about body configuration the control problem is greatly simplified because quiet stance can be modeled as a dual link inverted pendulum for both normal and labyrinthine loss subjects (53, 54), and for perturbed stance (55, 56). Consequently, when head orientation with respect to gravity is provided by the otolith organs, albeit a noisy signal (34), the center of pressure under the feet, approximately reflecting the body center of mass, can be regulated to control sway and prevent falling. Such cues can be profoundly important as shown by Rademaker's classic observations in Das Stehen (57) as well as many recent studies showing how standing on foam to degrade foot cues increases body sway, e.g., Creath et al. (55); Horlings et al. (53); Karmali et al. (34).

The advantage of hand contact for the labyrinthine-loss subjects is that a fiducial static reference is available by finger contact and can be used to drive an automatic pincer grip long-loop reflex, with the finger and feet serving as the pincer elements. It is also important to realize that what we typically refer to as “vestibular thresholds” are actually multisensory thresholds. To stimulate the otoliths or semicircular canals, forces have to be applied to the surface of the body to displace it to stimulate the vestibular receptors. Such forces necessarily activate a range of somatosensory afferents. This is why, under passive tilt conditions, seated vestibular loss subjects can be as accurate as normal subjects in indicating when they are aligned with the gravitational vertical. In passive tilt, somatosensory cues about pressure distribution on the body surface are adequate for them to perform well despite their absence of otolith function (58).

The oculogravic illusion refers to the apparent upward displacement of a visual target when a subject is exposed to a centripetal force causing a change in magnitude of the resultant gravitoinertial force vector. A subject facing the center of rotation and fixating a visual target will see the target displace upward and simultaneously experience backwards self-tilt (59). Labyrinthine loss subjects exhibit oculogravic illusions with similar time course but diminished magnitude relative to normal controls (60). Importantly, when control subjects and labyrinthine loss subjects are allowed to stand and align themselves with the gravitoinertial resultant vector during rotation there is no significant difference in the magnitude of the oculogravic illusions they experience (61). By contrast, when normal and labyrinthine loss subjects, while submerged in water to the neck to attenuate body contact cues, are exposed to centrifugation to generate oculogravic illusions, the normal subjects exhibit little decrement in oculogravic illusion magnitude but it is abolished or diminished greatly in magnitude for the subjects without vestibular function (62).

These studies with labyrinthine loss subjects show the multiple factors that can be affecting performance under normal conditions and that can easily elude notice. They emphasize the importance of body contact with the environment and the wide range of information obtained thereby. Additional insights into how postural control can be simplified are embodied in a new non-parallel engaged leg model of postural control that explains and predicts body sway patterns in stationary and rotating environments, and in hyper-gravity conditions (56, 63–67). The model shows how foot based control can simplify adaptive maintenance of upright balance.

### The Role of Velocity Storage During Dynamic Balance Control

In a classic paper, Cohen et al. systematically described the “velocity storage” of semicircular canal signals, with vestibular afternystagmus outlasting the peripheral time constant of the canals and of optokinetic afternystagmus outlasting optokinetic nystagmus (68). These observations are relevant to the inability of subjects exposed to vertical yaw and to horizontal roll rotation in our MARS device to track their ongoing position. They point to what Cohen would identify as a “leaky” integrator. Velocity storage is also an important factor in motion sickness evocation (69, 70). In our early parabolic flight studies, we had found that velocity storage of semicircular canal and optokinetic signals is greatly attenuated in weightless conditions. The time constant of post-rotation afternystagmus is ≈15 s in 1 g straight-and-level flight and ≈9.5 s in the weightless phase of flight (71, 72). This finding helped explain why Coriolis cross-coupling stimulation caused by head movements out of the axis of rotation is so provocative and nauseogenic in 1 g but was so mild when studied in the Skylab M-131 experiments conducted in orbital flight (73).

**Figure 15:**
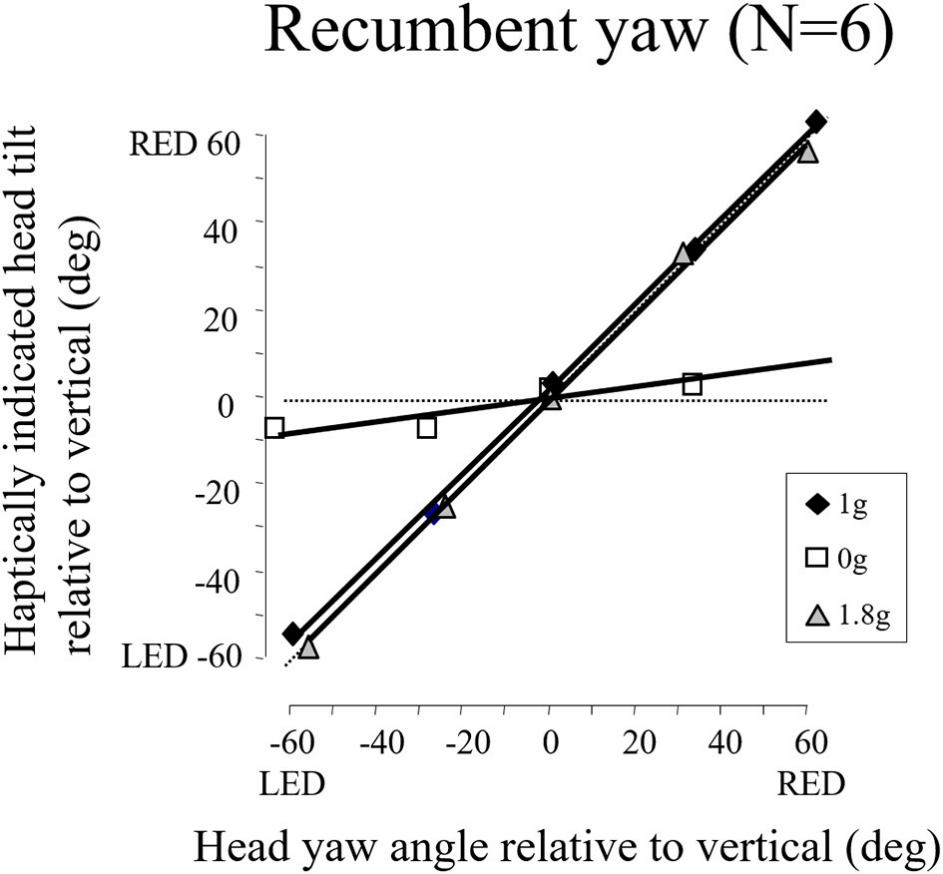
Recumbent subjects (*N* = 6) indicated the amplitude of imposed passive yaw-axis rotations during the 0 and 1.8 g phases of parabolic flight maneuvers and in straight-and-level flight. Rotations were 30° or 60° in amplitude and lasted <1.5 s. Subjects used a gravity neutral pointer and tried to keep it aligned with their start position. In 0 g, rotations were greatly underestimated. Similar results were obtained for vertical yaw axis rotation in 0 g.

We later found in the weightless phases of parabolic flight that when we exposed blindfolded subjects to angular displacements that were well above horizontal semicircular canal thresholds on Earth, they reported a slight tug on their body but underestimated or failed to detect their angular displacement (see Figure 15). The canal signals are not being accurately integrated in weightless conditions to give a sense of positional displacement (74). This finding is consistent with our observations that velocity storage is substantially attenuated in weightlessness. It is also consistent with the loss of a sense on one's ongoing position in the MARS supine roll and upright yaw dynamic balancing conditions, where the direction of balance cannot be sensed, only strategies to avoid crashing can be developed.

However, a puzzle remains: velocity storage is also attenuated in 1.8 g acceleration levels but head movements during rotation are greatly enhanced in provocativeness compared to 1 and 0 g acceleration levels (71, 72, 75, 76). Loading of the head by adding a mass to increase its weight can be provocative under 1 g conditions (77). Is this the causative factor for increased provocativeness of Coriolis cross-coupling in 1.8 g conditions (78)?

## Concluding Remarks

When Bernie Cohen invited me to participate in the meeting for which this paper has been prepared, he reminded me of all the NASA working groups we had been on together and the wonderful times we had. He asked me to put in context how my early NASA work had influenced my later work on human sensory-motor adaptation to the terrestrial force environment. Little did I know when first studying the effects of head movements on susceptibility to motion sickness that a chance observation would lead to an insight that would become a theme of study in my future work. The realization when I closed my eyes while freefloating, that I had lost my sense of spatial anchoring meant that body contact with the environment was profoundly important for orientation. Hand contact later turned out to be important as well for calibrating auditory and visual localization, for updating motor control, and even for influencing the perceived dimensions of the body (79).

## Author Contributions

The author confirms being the sole contributor of this work and has approved it for publication.

## Conflict of Interest

The author declares that the research was conducted in the absence of any commercial or financial relationships that could be construed as a potential conflict of interest.
